# Evaluation of physical activity among undergraduate students in Mogadishu Universities in the aftermath of COVID-19 restrictions

**DOI:** 10.7717/peerj.14131

**Published:** 2022-10-10

**Authors:** Sameer Badri AL-Mhanna, Wan Syaheedah Wan Ghazali, Mahaneem Mohamed, Abdulrahman Mohammed Sheikh, Abedelmalek Kalefh Tabnjh, HafeezAbiola Afolabi, Yahkub Babatunde Mutalub, Azeez Omoniyi Adeoye, Mariam Mohamed Nur, Monira I. Aldhahi

**Affiliations:** 1 Department of Physiology, School of Medical Sciences, Universiti Sains Malaysia, Kubang Kerian, Kelantan, Malaysia; 2 Faculty of Health Science, Somali International University, Mogadishu, Somalia; 3 Jordan University of Science and Technology Department of Applied Dental Sciences, Irbid, Jordan; 4 Department of General Surgery, Universiti Sains Malaysia, Kota bharu, Kelantan, Malaysia; 5 Department of Clinical Pharmacology, College of Medical Sciences, Abubakar Tafawa Balewa University, Bauchi, Bauchi, Nigeria; 6 Anatomy Department, Kampala International University, Bushenyi-Uganda, Bushenyi, Uganda; 7 Department of Rehabilitation Sciences, College of Health and Rehabilitation Sciences, Princess Nourah bint Abdulrahman University, Riyadh, Saudi Arabia

**Keywords:** Lockdown, Coronavirus disease, Exercise, Public health, Questionnaire

## Abstract

**Background:**

International restrictions were enacted during the COVID-19 pandemic to limit social interaction and viral transmission. These measures had a negative impact on physical activity (PA), creating changes in students’ health and lifestyles. The present study aimed to evaluate the levels of PA among undergraduate students in three different universities in Mogadishu after the relaxation of COVID-19 lockdown using the international physical activity questionnaire-long version (IPAQ-L) and its potential associated factors.

**Methods:**

This study is a multicentral study conducted at Somali International University, Horn of Africa University, and Daha International University. A total of 1,189 respondents were asked to answer the online questionnaire provided via a link shared using their social media.

**Results:**

After COVID-19 restrictions approximately ≥ 150 minutes of PA per week was reported by 500 men (97.3%) and 652 women (96.6%) at work. While 7 (1.4%) of men and 20 (3%) of women participate in < 150 minutes each week, respectively. Furthermore, only seven (1.4%) of males and three (0.4%) of women reported to have not performed any PA at work.

**Conclusion:**

The majority of the undergraduate students at the selected universities in Mogadishu were physically active after the relaxation of COVID-19 rules in Somalia. Such a high level of PA is a significant advantage to public health.

## Introduction

Physical activity (PA) is defined as any body movement resulting in energy consumption generated by the muscles which might be unplanned, and it can be a daily life activity, a workout that consists of programmed, intentional, and repetitive activity in grassroots and competitive sports and a regular physical activity of moderate intensity such as walking, cycling or sports that brings significant health benefits (Caspersen, Powell & Christenson, 1985). Physical inactivity is a global health problem that causes more than two million deaths each year, making it one of the top ten leading causes of death and disability (Organization, 2009). International restrictions were implemented during the coronavirus disease-2019 (COVID-19) pandemic to reduce socialisation and viral transmission (Ahmed et al., 2020; Al-Mhanna et al., 2021; Yusof et al., 2021). PA is negatively affected by these measures. Cross-sectional studies showed a significant reduction in PA levels during the pandemic, Ammar et al. (2020) conducted a study from April 6 to 11, 2020, among 1047 people from Africa (40%), Asia (36%), Europe (21%), and elsewhere (3%) participated in the worldwide survey. The later study found a significant reduction in PA due to COVID-19 restrictions (for example, a 24% reduction in the number of days/weeks of moderate-intensity PA and a 34% reduction in the number of minutes of walking per day) (McCarthy, Potts & Fisher, 2021).

However, there is strong evidence that regular PA is associated with significant health benefits and improved quality of life for individuals of all ages (Chodzko-Zajko et al., 2009). In contrast, limited PA or more concerning, the inability to engage in a regular PA as a result of strict quarantine may be linked to several other adverse metabolic effects that would dramatically increase the risk of a variety of severe and disabling disorders such as diabetes (Bhaskarabhatla & Birrer, 2005), cancer, (Al-Mhanna et al., 2022; Sanchis-Gomar et al., 2015) osteoporosis (Castrogiovanni et al., 2016), and cardiovascular disease (Lippi, Sanchis-Gomar & Therapy, 2020). In order to counteract the negative effects of these chronic diseases, several arrays of data have clearly shown that regular PA decreases the chance of acquiring multiple chronic illnesses and disorders thus, enhances life expectancy by lowering both cardiovascular disease and mortality (Al-Zoughool, Al-Ahmari & Khan, 2018; Fogelholm, 2010; Katzmarzyk, Janssen & Ardern, 2003; Oja et al., 2010). In the east, Hasan et al. (2021) reported that more physically active teenagers had a lower body mass index (BMI) than those who were less physically active. PA can help reduce body weight and BMI, enhancing musculoskeletal health and decreasing co-morbidity.

Currently, there is extensive literature that has evaluated the PA level, pre and during the pandemic-induced changes in people’s health and lifestyles (Lesser & Nienhuis, 2020; Maugeri et al., 2020; Paterson et al., 2021; Stockwell et al., 2021). A systematic review and meta-analysis involving 57 studies with a total sample size of 119,094 individuals from 14 countries worldwide, with participants ranging in age from 4 to 93 years. Only five studies indicated a substantial PA rise during the pandemic, while 32 studies found a significant decrease in PA levels. Fourteen studies yielded mixed results (Wunsch, Kienberger & Niessner, 2022). However, there is a lack of studies reported on PA among Somalian students (Persson et al., 2014). Also, the evaluation of PA has not been carried out among Somalian students after the COVID-19 restrictions. Students were denied the chance to perform PA due to the COVID-19 pandemic and nationwide lockdown. However, the physical distancing  policies implemented during COVID-19 and the suspension of on-site education might affect crucial social bonds and physical activity opportunities for students (Shepherd et al., 2021). Currently, there is a dearth of knowledge on the PA level of students after relaxation of COVID-19 restriction. The closest study available was that of Hurter et al. (2022) who assessed the PA level of students in Wales, United Kingdom upon return to school after easing COVID-19 restriction. There is no study to date that assessed the PA level of undergraduate students of Somalia after the relaxation of COVID-19 restrictions. The present study, therefore, aimed to evaluate the levels of PA among undergraduate students in three different universities in Mogadishu after the relaxation of COVID-19 lockdown using international physical activity questionnaire-long version (IPAQ-L) and its potential associated factors.

## Material and Methods

### Study design, populations, and sample

This is a descriptive cross-sectional multi-center study comparing measures of the last seven days of PA using IPAQ-L. The target populations were undergraduate students studying at three different universities in Mogadishu (Somali International University, Horn of Africa University, and Daha International University). Students filled out the consent form and voluntarily agreed to partake in the study. A total of 1,523 potential respondents were contacted *via* social media platforms using a simple random method from a list of registered students at respective universities, but only 1,189 responded to the request and agreed to participate in the study. All respondents that agreed to participate were then contacted *via* phone calls to confirm their participation and explain how to respond to the questionnaire. The respondents answer the online questionnaire provided *via* the social media platform. The questionnaires did not contain any identifier of the respondents to ensure confidentiality. The study was conducted between July 2021 to January 2022. The questionnaire contains responses to socio-demographic information like age, academic year, marital status, and gender. All the 1,189 students who agreed to participate in the study completed the questionnaires. The research was approved by the received institutional ethical approval from the ethics committee of Somali International University with approval number SIU/2021/RE/00096.

### Tools

With the WHO and the US Center for Disease Control and Prevention guidelines, a research team from several nations developed the IPAQ-L (Craig et al., 2003). The IPAQ-L is unique in that it evaluates all potential health-related PA that might occur in a variety of settings. The IPAQ-L is available and validated in two forms (along with 27 form items and a short seven form items) by using a 7-day reference period. The IPAQ-L examines five activity categories independently and offers information on PA intensity levels. IPAQ-L form is a PA evaluation tool used across the world (Craig et al., 2003).

### Measures

A modified IPAQ-L form was used to assess PA levels in five domains (transportation, work, housework, job, and leisure time). The only modification made to the original IPAQ-L was the substitution of school for work in the work domain of the form. This was because the participants were students. The IPAQ-L score was calculated as the sum of duration of moderate activity (in minutes) plus twice the duration of vigorous activity (in minutes) as used in the previous study (Hallal et al., 2003). The IPAQ-L defines moderate exercise as any activity lasting at least 10 min and resulting in a rise in heart rate, breathing rate, and/or perspiration production, while vigorous activities refer to any activity that causes a significant increase in breathing, heart rate, and perspiration. A domain-specific PA score was calculated as above for each domain of PA independently (at work, job, transportation, housework, and leisure). The total PA score was calculated by adding the scores for all the domains. In this study, a score below 150 min per week is regarded as physical inactivity, while a score of 150 min and above per week is regarded as physically active according to the recommendation of United States Department of Health and Human Services (PAGACR, 2008).

### Inclusion criteria

Registered undergraduate students who have access to the institutional social media groups as well as being able to speak English fluently.

### Exclusion criteria

The postgraduate students.

### Statistical analysis

Data were entered into SPSS software (IBM SPSS Statistics version 26 Armonk, NY USA). The data were first cleansed and checked for missing data. Outliers were tested using Tukey’s method, while normality was confirmed using the Kolmogorov–Smirnov (K-S) test. Descriptive analysis was represented as mean and standard deviation for numerical variables, whereas frequency with percentage was used for categorical variables. One-way analysis of variance (ANOVA) and independent *t*-test were used to explore the associations between PA and sociodemographic variables using a significant level of *p* < 0.05.

## Results

The final sample consisted of 1,189 students. Demographic data are presented in Table 1. On average, the age of men were 21.94 + 2.47 years, and women were 20.93 + 1.93 years. The majority of the students were in the fourth year of academic study, men (191 [37.2%]) and women (369 [54.7%]).

Total PA scores and activity scores for each activity regarding PA are shown in Table 2. The respondents are classified into three groups on the table: ≥ 150 min of PA per week, 1–149 min per week, and no self-reported PA. Considering the overall PA, all the respondents (100%) irrespective of their gender were physically active having at least 150 min of combined moderate and/or vigorous PA per week. The IPAQ evaluation also indicates that each participant’s PA domain has high levels of engagement. Approximately ≥ 150 min of PA per week was reported by 500 men (97.3%) and 652 women (96.6%) at work. While 7 (1.4%) of men and 20 (3%) of women participate in <150 min each week, respectively. Furthermore, only seven (1.4%) of males and three (0.4%) women reported having not performed any PA at work.

Self-reported PA levels for household PA were even more remarkable, with 468 (91.1%) men and a predictably higher number of 646 (95.7%) women indicating that they surpassed the 150-minute-per-week recommended from PA conducted while doing household. While only 46 (8.9%) of men and 29 (4.3%) of women reported <150 min per week performing PA doing household. According to IPAQ assessments, 511 (99.4%) men and 652 (96.6%) women reported 150 min or more of physical exercise per week in conventional leisure activities. Finally, concerning transportation-related PA, about 92% of both men and women reported at least 150 min of PA due to transportation-related PA per week.

Table 3 presents the comparison of PA outcome of respondents based on gender. The mean for the male was significantly higher than that of the female for the total job and transportation 1,233 *vs* 1,095 and 930 *vs* 813, respectively (*p* < 0.05). However, the reverse trend was noted on PA for “Housework”, the outcome failed to show a significant difference, although a higher mean was observed for the women 936 ± 745 than the men: 875 ± 727. Table 4 shows significant differences between respondents’ marital status and total PA as well as housework PA domain (*p* < 0.05) but not with a job, transportation, nor leisure PA domain (*p* > 0.05).

Table 5 shows a significant difference between the academic year groups of respondents and the PA of all the domains studied as well as the total PA (*P* < 0.05). The *post hoc* for multiple comparisons test revealed that the differences between total job and academic year groups were accounted for by differences between the late academic years group compared to the early and intermediate academic year group (*p* < 0.05). Also, the differences between total transportation and academic year were accounted for by the difference between the late academic year group compared to the intermediate year group (*p* < 0.05). Furthermore, the differences between academic year groups and total leisure time as well as total time were accounted for by the difference between all the groups (*p* < 0.05). However, the difference between the late and intermediate academic groups does not account for the observed significant differences between total housework time and the academic groups (*p* > 0.05).

## Discussion

This is the first research to report on the PA profile of Somalian students after the relaxation of COVID-19 restrictions using the IPAQ-L. The main finding is that most Somali students were adequately active, based on high levels of moderate and vigorous activity reported in the school and leisure domains. During the COVID-19 pandemic (Al-Wraikat & Ahmed, 2021), women and men are similarly affected by 30-day restrictions, with a significant decline in moderate and vigorous PA in both genders (Karuc et al., 2020). One explanation for the findings maybe an already existing high PA level among Somalian students. Countries of the world also reported a high level of PA as reported by a worldwide survey which found that around 89% of the respondents met the required level of physical exercise in many countries (Sáez, Solabarrieta & Rubio, 2021).

The reason for the finding of the present study can probably be explained by the assumption that after returning to their respective universities (Somali International University, Horn of Africa University, and Daha International University), students might be highly motivated to participate in PA (DHSC, 2019). When upper primary school students returned to school, 50.8% of them fulfilled the UK government’s moderate and vigorous PA guidelines of 60 min each day (UKG, 2021). However, a Brazilian study using the IPAQ short-form instrument found that 41.1% of Brazilian individuals aged 20 and above are inactive (Macera et al., 2003). Although the task of comparing inactivity prevalence across countries is difficult due to differences in survey sampling and assessment methods, the current estimate of high PA level found in the current study appears to be different from that reported in previous research using different measures of PA. According to data from the Behavioral Risk Factors Surveillance System in the United States, the majority of individuals in the United States (54%) are not physically active enough to fulfil the recommended guidelines of at least 30 min of moderate-intensity exercise most days of the week (Abrantes, Lamounier & Colosimo, 2003). There is little evidence to support the claim that Somali students are much more active than students in other developed and developing countries concerning resolving the various theories for the findings. There is evidence to show that the high levels of PA reported by the students are unlikely to be accurate representations of their actual levels of PA (Brasil, 2012) .

Lack of PA is a well-known risk factor for cardiovascular disease, which is the world’s fourth major cause of death worldwide, Devlin et al. (2012) showed that in Somaliland, a high level of physical inactivity and obesity might become a major public health concern. Obesity (BMI 30 kg/m^2^) was found in 44% of Somali women in Norway and 31% of Somali women in Somaliland, respectively (Ahmed et al., 2018). Long-term preventative methods are required, based on chronic disease prevalence statistics from the above-mentioned studies. However, we cannot explain why a randomly chosen representative sample of Somali students reported being much more active. In this study, students had considerable difficulty evaluating their PA, and this is a cause for concern since the amount of PA involvement is not well understood. Students tend to have difficulties conceiving and distinguishing between concepts like “moderate PA” and “vigorous PA,” as well as understanding what a 10-minute session of PA implies (Matsudo et al., 2001). Hence students’ estimation of moderate and vigorous PA at school and home seems to be the most difficult areas in the present research.

Despite the high levels of self-reported PA in the current study, the COVID-19 pandemic has impacted unexpected disruption to the student’s lifestyles. Most countries implemented COVID-19 restrictions to prevent the virus’s spread (Ahmed et al., 2022; Ahmed et al., 2020). Schools, sports clubs, and indoor fitness centres, including swimming pools, were closed, and students no longer had access to school-based PA. Furthermore, all scheduled leisure activities ceased due to lockdown restrictions preventing people from assembling even in open places. As a result, recent systematic and scoping reviews found a substantial decrease in PA levels as compared to pre-pandemic levels in the majority of studies included (Stockwell et al., 2021). Specifically, self-reported PA levels dropped by 91 min per day in 113 Spanish populations (Medrano et al., 2021). Compared to pre-pandemic levels, 36% of parents of 211 children ages 5–13 in the United States indicated their children had done much less PA (Dunton, Do & Wang, 2020). Regardless of these facts, it has been observed that PA levels increased significantly upon returning to school when the restrictions were reduced (Hurter et al., 2022). All the aforementioned facts might explain the high level of the reported PA in the current study after reducing the restrictions in the respective universities.

On the other hand, the environmental factors may enhance PA engagement. Somalia is known for having Africa’s longest coastline, which is located in the Horn of Africa and having primarily flat land, making it appropriate for PA engagement. Additionally, the demographic presentation in Table 1 indicated that most of the students were aged 20 years and in the fourth year of academic study. However, physical inactivity was more common among those with lower levels of education in the United States (Crespo et al., 1999). It is not surprising that physical inactivity increases as people become older. PA declines with age, documented in the literature (Caspersen, Pereira & Curran, 2000; Ham et al., 2004). According to a survey of Nigerian civil servants, there was no significant PA trend between the ages of 20 and 64 (Forrest et al., 2001). Meanwhile, according to data from the recent Behavioral Risk Factors Surveillance System research in the United States, physical inactivity gradually increased with age in both sexes, from 18–29 years to 70 years (Forrest et al., 2001). In this study, most of the students were 20 years, highlighting the importance of high levels of PA for undergraduate students in Somalia to maintain health.

**Table 1:** Participants demographic description (*N* = 1189).

	Men (*n* = 514)	Women (*n* = 675)
Age, years, mean (SD)	21.94 (2.47)	20.93 (1.93)
Academic year, *n* (%)		
1	32 (6.2)	16 (2.4)
2	95 (18.5)	100 (14.8)
3	102 (19.8)	129 (19.1)
4	191 (37.2)	369 (54.7)
5	34 (6.6)	28 (4.1)
6	60 (11.7)	33 (4.9)
Marital status, *n* (%)		
Single	434 (84.4)	591 (87.6)
Married	80 (15.6)	76 (11.3)
Widow	0 (0)	8 (1.2)

**Table 2:** Self-reported physical activity using the International Physical Activity Questionnaire (*N* = 1189).

	**Men (*n* = 514)**	**Women (*n* = 675)**
**PA total, *n* (%)**		
≥ 150 min/week	514 (100)	675 (100)
1–149 min/week	–	–
None	–	–
**PA work, *n* (%)**		
≥ 150 min/week	500 (97.3)	652 (96.6)
1–149 min/week	7 (1.4)	20 (3)
None	7 (1.4)	3 (0.4)
**PA household, *n* (%)**		
≥ 150 min/week	468 (91.1)	646 (95.7)
1–149 min/week	46 (8.9)	29 (4.3)
None	–	–
**PA leisure time, *n* (%)**		
≥ 150 min/week	511 (99.4)	664 (98.4)
1–149 min/week	3 (0.6)	11 (1.6)
None	–	–
**PA transportation, *n* (%)**		
≥ 150 min/week	483 (94)	621 (92)
1–149 min/week	31 (6)	39 (5.8)
None	–	15 (2.2)

**Table 3:** Gender characteristics of physical activity engagement (*N* = 1189).

**Characteristics**	**Women (*n* = 675)**	**Men (*n* = 514)**	**T stat**	***P*-value**
	**Mean (SD)**	**Mean (SD)**		
Total Job	1095 ± 778	1233 ± 835	2.933	0.003^*^
Total Transportation	813 ± 652	930 ± 675	3.027	0.003^*^
Total House Works	936 ± 745	875 ± 727	1.402	0.16
Total Leisure Time	1001 ± 777	1042 ± 719	−.925	0.35
Total	3845 ± 1730	4081 ± 1799	2.284	0.02^*^

**Notes.**

* Significance level set at *p* ≤ 0.05; Independent *t*-test was used for statistical analysis.

**Table 4:** Comparing means of physical activity according to marital status (*n* = 1181).

**Characteristics**	**Single** ***N* = 1025**	**Married** ***N* = 156**	**T stat**	***P*-value**
	**Mean (SD)**	**Mean (SD)**		
Total Job	1138 ± 801	1223 ± 776	−1.237	0.21
Total Transportation	854 ± 670	927 ± 600	−1.287	0.19
Total House Works	885 ± 717	1084 ± 846	−2.786	0.006^*^
Total Leisure Time	998 ± 681	1149 ± 1102	−1.667	0.09
Total	3877 ± 1743	4385 ± 1849	−3.365	0.001^*^

**Notes.**

* Significance level set at *p* ≤ 0.05; Independent *t*-test was used for statistical analysis.

**Table 5:** Comparing means of physical activity according to academic level groups (*N* = 1189).

**Characteristics**	**Early** **(1** ^**st**^ **& 2** ^**nd**^ **year)** ***n* = 243**	**Intermediate (3** ^**rd**^ **& 4** ^**th**^ **year)** ***n* = 791**	**Late** **(5** ^**th**^ **&6** ^**th**^ **year)** ***n* = 155**	**F value**	***P*-value**
	**Mean (SD)**	**Mean (SD)**	**Mean (SD)**		
Total Job	1097 ± 822	1145 ± 793	1294 ± 827	3.038	0.048^*^
Total Transportation	856 ± 755	840 ± 625	988 ± 692	3.252	0.039^*^
Total House Works	764 ± 538	931 ± 762	1026 ± 841	9.677	0.000^*^
Total Leisure Time	885 ± 554	1018 ± 734	1230 ± 1019	9.229	0.000^*^
Total	3603 ± 1619	3935 ± 1740	4540 ± 1945	12.528	0.000^*^

**Notes.**

* Significance level of the main effect set at *p* ≤ 0.05; One-way ANOVA Test was used for statistical analysis.

A more reasonable explanation for the current research finding is that the students systematically overestimated their moderate and vigorous PA levels, which might be attributed to IPAQ-L interpretation issues (Hallal et al., 2010). Similarly, the Brazilian version of IPAQ-L overestimated self-reported PA outcomes (Sebastiao et al., 2012). Male and female respondents in this stratified random representative sample (*n* = 1572) exhibited unusually high levels of PA in-home and work-related domains, although without direct measurements (Sebastiao et al., 2012). Overall, they had remarkably high results, with 83% of men and 89% of females exceeding the 150-minute-per-week moderate-to-vigorous PA level, raising questions about using the Brazilian version of IPAQ-L only for community health plans (Barnett et al., 2007). Inconsistent findings among older people in the United Kingdom (age 71.8 ± 6.6 years) reported that they underestimated their level of moderate-to-vigorous PA and sitting time (Cleland et al., 2018). A 165% overestimation of total PA was also found during the validation study of the IPAQ-L in New Zealand (Boon et al., 2010). Johnson-Kozlow et al. (2006) illustrated that, when compared with accelerometer measurements, the IPAQ underestimated overall PA by 247%. In a comprehensive review, Lee et al. (2011) reported on three studies (Dinger, Behrens & Han, 2006; Macfarlane et al., 2007; Timperio et al., 2004) that overestimated metabolic equivalent tasks on PA by 101–173% min/week for US, Chinese, and Australian populations using IPAQ-short form. However, these overestimations of the pre-pandemic self-reported PA findings are in line with the present research, especially when the COVID-19 restriction was relaxed. Also, the high number of minutes spent performing in the PA recorded in this research might be attributed to the large proportion of students

Many recent research findings have stated that the IPAQ is linked to a significant overestimation of PA levels in various populations (Johnson-Kozlow et al., 2006; Pitta et al., 2006). Hallal et al. (2010) identified issues with over-reporting and problems assessing levels of PA using the IPAQ-long form. Males were much more likely than females to report unexpectedly high levels of PA at work (men $ \frac{1}{4} $ 1564 min per week; women $ \frac{1}{4} $ 1157 min per week), and females were more likely to report unexpectedly high levels of PA in household activities (average $ \frac{1}{4} $ 710 min per week). Both men and women reported values that far surpassed the recommended amount of PA (150 min per week) in practically every aspect. Hallal et al. (2010) found various issues with the IPAQ-long form in a recent review study, including serious issues with measuring PA levels. From the study’s outcome, the student showed a very high level of PA, which is probably due to the high enthusiasm for their returning to school and loosening of the COVID-19 rules. The students returned to colleges with enthusiasm, knowing that the COVID-19 rules had been relaxed. Hence, they were excited to participate in a high level of various PA.

In this research, males reported spending more time on work, leisure, and transportation PA than females. While women reported higher levels of PA in the household compared to males. In another research, women reported higher PA levels at work and comparatively less leisure-time PA (Pomerleau et al., 2000). According to the majority of past research, males are more active than females (Forrest et al., 2001; Hallal et al., 2003; Ham et al., 2004; Martin et al., 2000). This study has several strengths. It is the first study to use the IPAQ-L among Somalin students after the COVID-19 restrictions. This study also used a wide range of different universities students’ participation involving a large sample size to evaluate the PA level. Concerning limitations, the sample distribution revealed that the study had more women respondents than men which may influence the generalizability of the finding. The study did not highlight the specific types of PA that contributed most to the high PA outcome, which might add valuable information about the most commonly reported PA. Furthermore, the likelihood of overestimating the physical activity by the respondents is a concern.

## Conclusion

This study demonstrated that undergraduate students in selected universities in Mogadishu were physically active after the relaxation of COVID-19 rules. Such a high level of PA is a significant advantage to public health, especially after COVID-19 restrictions were relaxed. Therefore, public policies are needed to maintain an active lifestyle and discourage sedentary habits among undergraduate students of Somalia. Healthcare providers have an important role in promoting PA among the populations. Furthermore, because the long-term impacts of the COVID-19 pandemic are uncertain, longitudinal research is needed to investigate the associations between PA, mental health, and well-being among undergraduate students in Mogadishu after the relaxation of COVID-19 restrictions.

## Recommendation

Somalia Universities should maintain the school policies that encourage an active lifestyle among the students in order to sustain and/or improve the current level of PA.

##  Supplemental Information

10.7717/peerj.14131/supp-1 Supplemental Information 1 Raw data Participants’ demographic description, self-reported physical activity using the International Physical Activity Questionnaire and gender characteristics of physical activity engagement.

## Additional Information and Declarations

### Data Availability

The following information was supplied regarding data availability: The raw measurements are available in the Supplemental Files.
